# Teacher Perspectives on Primary-Secondary School Transition Projects During the COVID-19 Pandemic

**DOI:** 10.1177/21582440231181382

**Published:** 2023-06-16

**Authors:** Samson Maekele Tsegay, Lucie Wheeler, Phil Kirkman, Simon Pratt-Adams

**Affiliations:** 1 Anglia Ruskin University Cambridge, UK

**Keywords:** teacher perspectives, school transitions, opportunity area, student resilience, COVID-19

## Abstract

Opportunity areas are primarily selected to improve the social mobility of citizens using education. This paper explores teachers’ perspectives on school transitions, particularly emphasizing the role of school transition intervention activities in supporting students’ resilience, behavior, academic understanding, and positive parental involvement. Informed by Multiple and Multi-dimensional Transitions (MMT) theory, the paper focuses on the outcomes of school transition intervention activities applied to new Year 7 students in a UK opportunity area. Data was collected through document review, teacher survey, and semi-structured interviews. As a result, 14 interventions were identified, such as a summer school program, peer mentoring, and interschool visits, aiming to make primary to secondary school transition smoother. However, the findings suggested that many schools did not employ some of the school transition intervention projects. Moreover, the data indicated that the COVID-19 pandemic negatively affected the implementation of many of the school transition projects. The paper contributes to understanding the impact of school transition projects on students’ confidence, wellbeing, and academic achievement.

## Introduction

School transitions, or transfer of students from one school to another, is an important period in the lives of children and their families (Bagnall et al., 2022; Sutherland et al., 2010). Although we use school transition and transfer interchangeably to indicate the movement of students from one school to another, we acknowledge the different uses of transfer and transition. For instance, Galton et al. (2003) defined transfer “as the move from one school to another” and transition “as the movement of pupils to a new class at the end of each academic year, within the same school” (p. 1). This paper adopted Divya Jindal-Snape’s definition that school transition is a dynamic and ongoing process of educational, social, and psychological adaptation over time due to changes in context, interpersonal relationships, and identity (Jindal-Snape, 2018, 2020). The transfer to secondary school means that students need to adapt to a different and, perhaps, more challenging school environment (Gilbert et al., 2021; Spernes, 2022). These challenges are often related to new or different academic structures and requirements as well as social interactions with students and teachers (Jindal-Snape, 2018; Nowland & Qualter, 2020; Rice et al., 2015). Studies on school transition suggest that students require relevant support mechanisms to adjust to new schools and make a successful transition (Chambers & Coffey, 2019; Jindal-Snape, 2018)

A range of personal, parental, and school-related factors affect students’ school transitions (Gilbert et al., 2021; Harris & Nowland, 2021; Roberts, 2015). In addition, Jindal-Snape (2018) adds that socioeconomic, demographic, and peer-related factors should be considered in analyzing students’ transition from primary to secondary schools. These factors have the potential to either facilitate or hinder the smooth and inclusive transition of students from and within schools. Moreover, school transition is a time of change, building resilience, and negotiating new environments and social relationships (Cartmell, 2011; Roberts, 2015). It can also affect students’ socio-emotional and academic performances positively or negatively, depending on the students’ experiences (Roberts, 2015; Weiss & Baker-Smith, 2010).

Although school transition involves some degree of apprehension for some students (Evangelou et al., 2008; Spernes, 2022), others find it to be an “exciting time of new experiences and widening horizons” (Sutherland et al., 2010, p. 11). Hence, it is both an opportunity and challenge for children and their parents and carers, as well as the school. Above all, the level of support the children get affects their school transition experiences, which have a long-term impact on their behavior, social adjustment in other settings, sense of security, and academic success (Cartmell, 2011; Smyth & Privalko, 2022).

Successful school transition requires coordinated efforts from stakeholders, particularly parents, students, and teachers (Akos & Galassi, 2004; Chambers & Coffey, 2019). Overall, students can be supported in many ways to lead and experience a positive transition, ensuring academic and behavioral involvement, and feeling a sense of belonging to their school. Suggestions for support include preparation of transition toolkits, peer support, summer visits, and summer schools (Galton et al., 2003). Furthermore, schools and teachers, in particular, often take the leading role in most of these activities, including school visits (Akos & Galassi, 2004; Cuconato et al., 2015). For example, in the inter-school visits, secondary school teachers can identify any curriculum gaps or unfinished projects and explore ways of building links and continuity to complete the work projects that students began in primary school (Rice et al., 2015).

This paper, therefore, explores teacher perspectives on school transitions, with a particular emphasis on the role of school transition intervention activities in supporting students’ resilience, behavior, academic understanding, and positive parental involvement. This paper is drawn from a broader research project conducted in a UK opportunity area based on data collected from secondary school teachers. It is guided by the following three overarching research questions: (1) What are the qualitative nature of the school transition intervention activities applied to Year 7 students? (2) What are the impact of the school transition intervention activities on Year 7 students? (3) What are the factors affecting the school transition in Year 7 students? The paper contributes to understanding the impact of school transition projects to improve students’ confidence, wellbeing, and academic achievement.

## Literature Review

There is a connection between school transition and students’ emotional and psychological wellbeing (Bagnall, 2020; Gilbert et al., 2021; Jindal-Snape, 2016; Spernes, 2022). A sound transition experience promotes students’ wellbeing (Jindal-Snape, 2016; Schlossberg et al., 1995). Transitions can also damage psychological wellbeing, but despite that, not many interventions focus on emotional resilience, making emotional interventions significant (Bagnall, 2020). Most children experience hormonal changes (Spernes, 2022) and face difficulty adjusting to a new school setting and new social groups, with lower self-esteem being a significant contributing factor in those experiencing poorer transitions (West et al., 2010). Building social relationships with other students and teachers is crucial for a school’s community (Spernes, 2022), and, arguably, good communication between schools helps build that sense of community (Coffey, 2013).

Research indicates that transition difficulties are associated with poorer levels of academic achievement, which could also have a long-term impact on the students’ socio-economic status (Smyth & Privalko, 2022). Academically, a temporary pause (Galton et al., 2000) or even regression (Arthur et al., 2010; Pietarinen, 2000; Weiss & Baker-Smith, 2010) in academic progress might occur within the year after the transition (Weiss & Baker-Smith, 2010). Evidently, this dip in attainment is documented in multiple countries for various reasons, such as parents’ support (Gilbert et al., 2021; Smyth & Privalko, 2022) and changes in the school environment (Boone & Demanet, 2020). However, students can be supported in various ways to lead a positive transition that ensures academic and behavioral involvement and a sense of belonging to their school (Galton et al., 2003; Jindal-Snape, 2016).

Schools, in collaboration with their partners such as the Department of Education, often use transition toolkits or resources to ensure an inclusive and successful transition (Evangelou et al., 2008). These resources include school transitions booklets, School Transition and Adjustment Research (STAR), and other related questionnaires. The school transition booklets which consist of the school environment, resources, and activities are used to prepare students for change (Bagnall et al., 2022; Yanık Özger, 2022). Whereas the STAR and other questionnaires are administered to teachers, students, or parents in secondary schools in order to identify students’ and parents’ key concerns, predict students’ adjustment, and assess school interventions used to support students, including those with special education needs (Rice et al., 2015). Transition resources provide students with adequate information about their new schools. They also equip parents with the necessary knowledge and attitude to support their children’s transition to secondary school.

LaBahn (1995) states that successful parental involvement in school and students consists of two interconnected points: active participation and commitment. With active participation in schools and commitment to improving their children’s experience, parents can support their children’s smooth transition and help them accomplish their goals (Spernes, 2022). There is extensive literature supporting the positive impact of parental engagement on students’ learning outcomes (Gilbert et al., 2021), but as students enter high school, parental engagement significantly changes (Mac Iver et al., 2015; Muller, 2005). This change in engagement may be due to parents’ beliefs that their children need to be more autonomous or their difficulty in helping with homework and explaining complex curricula (Simon, 2004; Smyth & Privalko, 2022).

Moreover, schools have different academic and social environments depending on various factors such as their ethos, resources, location, and the community around them (Nowland & Qualter, 2020). Therefore, school visits and related events such as summer schools can help parents and students understand the school's conditions and make relevant preparations for the students’ transitions. Such events often assist the students in familiarisation with their future schools and teachers and forging new friendships (Harris & Nowland, 2021). They also help the children (and their parents) to select schools that match their needs and interests. In addition, the inter-school visits help secondary school teachers to identify any curriculum gaps or unfinished projects and explore ways of building links and continuity to complete the tasks that students began in primary school (Rice et al., 2015).

## Theoretical Framework

This paper explores teacher perspectives on school transitions and provides a better understanding of the multiple and multi-dimensional educational transitions experienced by year 7 students. In particular, this paper focuses on teacher perspectives regarding the role of school transition intervention activities to support students’ resilience, behavior, and academic understanding and positive parental involvement. Hence, the paper adopted the Multiple and Multi-dimensional Transitions (MMT) theory, which affects the child and the significant others in the child’s ecosystem, such as their teachers and family (Jindal-Snape, 2020; Jindal-Snape & Cantali, 2019). The MMT theory advocates that transitions are not linear and sequential, but multiple and synchronous (Jindal-Snape, 2016), allowing people to experience different transitions at the same time, in multiple domains and multiple contexts (Jindal-Snape, 2018, 2020; Jindal-Snape & Cantali, 2019; Jindal-Snape et al., 2021). MMT theory also argues that one person’s multiple transitions (e.g., academic and emotional) impact each other and can trigger transitions of significant others (such as friends or parents) and vice versa (Jindal-Snape, 2016; Jindal-Snape & Cantali, 2019).

Furthermore, through MMT theory, this paper aims to develop a comprehensive understanding of the “interactions between complex multiple and multi-dimensional transitions” (Jindal-Snape et al., 2019, p. 3). As indicated above, school transition is a multifaceted and dynamic process influenced by multiple personal and institutional factors (Jindal-Snape, 2018, 2020). Personal characteristics and inner strengths influence how students adapt or transform their ecosystem and experience transition, making transition experiences different and complex for different students (Jindal-Snape et al., 2020). However, transition support, that students get from their significant others, could help them ease the risk factors and minimize the challenges they face during their school transition, leading to a positive transition experience (Jindal-Snape & Cantali, 2019; Rice et al., 2015). Jindal-Snape (2016) and Schlossberg et al. (1995) further argue that school and family support facilitates students’ school transition and promotes their social and emotional wellbeing. There is a correlation between school transition, and emotional adjustment and wellbeing. School transition is often associated with an increased level of stress (Bagnall, 2020), which, in turn, is associated with negative health outcomes, such as mental health problems (Roberts, 2015). Moreover, emotional regulation, including the ability to regulate distressing emotions, is associated with positive adjustment and wellbeing (Finkelstein-Fox et al., 2018).

## Methodology

### Research Context

This paper is drawn from a broader evaluation of primary to secondary school transition activities in a UK opportunity area during the COVID-19 pandemic. The effect of COVID-19 pandemic has been enormous in many sectors including education (Holt & Murray, 2022). With the spread of the COVID-19 virus and, hence, the increased mortality around the world, many countries temporarily closed their schools (Schleicher, 2020) or changed their teaching mode to online learning (Di Pietro et al., 2020). Similarly, from 2020 to 2021, the UK introduced lockdowns and other measures to control the spread of the virus. This has affected the teaching and learning process and students’ experiences of their school transitions (Bagnall et al., 2022). This paper specifically focuses on teachers’ perspectives on school transitions, with data collected through online survey and semi-structured interviews between June and July 2021. The paper draws on the original analysis and rethemes the data in order to address the research questions.

The Greater London Authority (2015) defines opportunity areas as “places where change and growth can happen.” They are social mobility “cold spots” which are identified by the UK Government for their potential for future improvement or development over a period of time (Cambridge City Council, 2012; Department for Education, 2016). Opportunity areas are primarily selected to improve the social mobility of citizens using education (Department for Education, 2017) as a key driver to ensure that all children have the opportunity to achieve their full potential whilst at school and college, and become well-equipped to make an informed decision about their future as adults (Cambridge City Council, 2012; Department for Education, 2016).

This paper is based on a research project conducted in 1 of the 12 opportunity areas in England (Department for Education, 2017). The target of the research project was to identify and analyze the impact of the new transition projects by a retrospective evaluation of the projects in the academic year 2019 to 2020 and an evaluation of the projects for 2020 to 2021. The transition projects involve pupils from Year 6 and Year 7 as they move between different schools in the transition from primary to secondary level. The projects are set against a background of low social mobility and higher than the national average of free school meals. The transition projects aim to target and support Year 7 students with poor resilience and behavior, limited academic and behavioral understanding of their new secondary schools, and limited parent/carer involvement as stakeholders in the transition process.

### Data Collection

The study adopted a mixed methods approach to provide stronger evidence to answer the research questions (Creswell & Plano Clark, 2011). Data for the study was collected using document review. Moreover, teacher survey and semi-structured interviews were conducted with 20 secondary school teachers having various roles associated with Year 7 students, such as subject teachers, principals, and pastoral managers.

#### Document Review

Document review was used to examine and describe each project/intervention. We reviewed school transition documents from online sources and documents provided by the sample opportunity area transitions team. In particular, documents related to students’ resilience, behavior, and academic understanding, and parental involvement were reviewed to get a better understanding of the background knowledge of the transition projects. This also fed into our project descriptions and design of the survey and interview questions. In general, we reviewed over 342 documents relating to the projects and transitions project schools.

#### Questionnaire Survey

Teacher survey (*n* = 20) was used to explore their involvement in the different projects and the extent to which they felt the projects were successful. The survey aimed to gain insight into the impact of transition projects on pupil outcomes. The survey was made available on JISCMAIL (a user-friendly online platform), with a call circulated to all the teachers with roles associated with Year 7 students. The survey included both open and closed-ended questions and was kept short, with a completion time of under 15 minutes, to encourage teachers’ participation. Some of the topics covered include teachers’ perception of barriers to transitions, factors affecting the school’s capacity to support the Year 7 students, and the impact of the projects both before and after implementation. Teachers were also asked to give their perspectives on the role of transition projects in students’ school transition before and during the COVID-19 pandemic.

#### Semi-Structured Interviews

We conducted semi-structured interviews with 20 teachers to probe the findings from the survey and gain a broader understanding of the teachers’ perspectives on the transition intervention activities and their impact on students’ transition. Purposive sampling was used to select participants with significant features for the study (Silverman, 2013), particularly those who could give us adequate information on the key subjects concerning previous and current transition projects. Accordingly, teachers with at least 1 year of experience in their present school were selected from different schools (six schools) and roles to get relevant information and develop a comprehensive understanding of the issue. Similar topics to those covered in the survey were asked in the interview, but with greater focus and depth. The interviews were conducted online through Microsoft Teams. The interviews were recorded with the participant’s permission, and the interview process took about 40 minutes.

### Data Analysis and Ethical Consideration

This paper applied a similar data analysis method to the original report: descriptive statistics and thematic analysis. However, in this paper, the data was narrowed down, rethemed, and reanalyzed to address the research questions. Descriptive statistics was used to analyze the survey data. The data from the closed-ended questions were summarized and described using frequency statistics, particularly percentages, to understand its content (Mishra et al., 2019). Moreover, thematic data analysis was employed to analyze the data collected through survey (open-ended questions) and semi-structured interviews to summarize the key features and offer a rich interpretation of the data (Braun & Clarke, 2006; Bryman, 2008). In particular, we applied Braun and Clarke’s (2006) six phases of conducting thematic analysis: familiarize with the data, generate initial codes, search for themes, review themes, define and name themes, and write-up.

The study followed BERA’s ethical guidelines. In addition, ethical approval to conduct the study was obtained from Anglia Ruskin University. Participants were provided with a consent form containing information about the research project, their permission to participate in the study and their rights to withdraw at any stage of the research. Specific attention was also given to confidentiality and anonymity by using pseudonyms or codes throughout the research process.

## Findings and Discussion

### School Transition Intervention Projects

Various school transition intervention activities were applied to new Year 7 students in the sample opportunity area. These projects were designed to improve students’ transition experiences and support them to be safe and confident, which affects their personal, social, and academic development and self-esteem (Department for Education, 2016). In Jindal-Snape’s (2016) explanation, the projects were aimed to allow students to experience different transitions, in multiple domains (Table 1).

**Table 1.** Available School Transition Projects.

Project	Description
The bridging project	Communication and running programs between the primary and secondary schools and curriculum, respectively, to develop continuity and development.
Child protection online management system (CPOMS)	Safeguarding software package for schools.
Common transfer document (CTD)	An excel sheet with key information about the child.
Interschool visits	School visits that help parents, students, and teachers to understand the conditions of students and make relevant preparations for their transitions.
School information booklets	Information booklets which are used to run an information event or transition induction before and after students’ admission.
Summer schools	One to two weeks summer program which help students to get experience and support about the academic and pastoral level of the new school and, thus, gain confidence and skills for secondary school transitions.
School transition and adjustment research (STAR) survey	Questionnaires administered to teachers, students, or parents in secondary schools in order to identify students’ and parents’ key concerns, predict students’ adjustment, and assess school interventions used to support students including those with special educational needs
Peer mentoring	A training course run by Essex Community CIC whereby older students were trained to support younger, targeted, students.
Parent information evenings	An open evening held at a community center. The purpose of this event was to help parents explore different secondary schools and meet secondary school headteachers.
Emotional literacy support assistants (ELSA)	Training provided for early intervention support. The course covers social skills, counseling skills, emotions, and self-esteem
Special educational needs and disabilities (SEND) training	A training package around SEN for school staff. This was delivered online via webinars.
Young minds training	A course offered by Young Minds to train teachers to provide mental health support. It involves understanding and developing (teaching) resilience in school settings.
Transitions week	A streamlined transition visit period between primary and secondary schools where children are able to visit the school and participate in activities/lessons.
Transition working group	A group set up to bring together stakeholders and to develop and implement strategy around transition work.

The paper suggested that these intervention activities were essential to students’ transition to secondary school. However, in most cases, their implementation was hindered, and their effectiveness decreased during the COVID-19 pandemic. As can be seen in Appendix 1, most of the teachers fell into the “I don’t know” category regarding the support of the transition projects during the COVID-19 pandemic, which suggests a lack of information due to the COVID-19 lockdown.

### Impacts and Influential Factors

As indicated above, school transition interventions are designed to facilitate successful transitions between primary and secondary schools. Similarly, the transition projects in the sample opportunity area enhanced students’ experiences by improving their resilience, behavioral and academic understanding, and enabling positive parental (carer) involvement. However, many schools did not employ some of the school transition intervention projects due to COVID-19 and other reasons. In this section, we have categorized the transition projects into three themes based on the nature of the activities and the primary expected outputs. Nonetheless, as seen below, it is important to note that many of the transition projects are interdependent and interconnected.

#### Transition Information Toolkits and Evening

These school transition projects aim to assess, identify, and share or transfer information. They mainly include CTD, CPOMS, information booklets, parent information evening, and STAR Survey. In addition to information sharing and time-saving, these toolkits also provide additional benefits which facilitate positive school transition. For instance, the survey data indicated that CTD is significant for grouping/banding, identifying vulnerable students, and providing data consistency. In contrast, CPOMS allows for safeguarding Special Education Needs (SEN) students, logging behavior, and maintaining parental contact.

The data showed that CTD is an essential tool for consistent data transfer, mainly if the primary school designed the data system in collaboration with the secondary schools. The spreadsheet document helps schools log quantitative and qualitative data about students, including achievement data, attendance data, information on friends, hobbies/interests, SEN, behavior, mental health support needs, safeguarding concerns, and medical history. The document also contains a comments section where the primary school teacher can add additional information about each child. All primary schools are signed up to use the spreadsheet document; thus, information is easily systematically transferred to the secondary schools. As SF-HS expressed, CTD “helps to build a picture of the child” with all those data. JS-LP also said:

“I’m much happier that it’s in one format because it’s much better than being emailed eight different things and trying to work out what goes on what. But I think it needs a lot of refining. I think the idea behind it is fabulous, I really do. But it needs some refining.”

In line with JS-LP’s statement, other participants suggested that other schools (outside of the sample opportunity area) should have been included in the CTD; but only schools in the sample opportunity area were part of the CTD. So, if a feeder school is not part of the CTD, schools depend on a multi-document approach for sharing students’ information. This suggests the need for a centralized database across all schools.

Regarding CPOMS, the interview findings suggested that the safeguarding software package is expanded beyond safeguarding to be used for information sharing from primary to secondary schools. In addition, the teachers found CPOMS useful in reducing paperwork. Nonetheless, they were concerned about expanding the document to include more information than the safeguarding as it could result in a breach of confidentiality. They argued that children should be given an opportunity for a fresh start in behavior at the new school. Hence, schools should use the document for its intended purpose (i.e., safeguarding). Moreover, the participants noted that CPOMS could not replace face-to-face interactions but can supplement it.

“I just think it’s a very useful system really!” (DE-GW).

“Secondary school can be a useful fresh start for a child which can sometimes be hampered by teachers sharing stories about their family, which can act as baggage in the new school.” (AB-SP).

“It doesn’t replace those face-to-face conversations as well, so we’re still very much having those conversations and people, you know members of staff, will still come and speak to people and put it on CPOMS so it’s not replacing it but it’s supplementing it.” (EM-MC).

The above excerpts show that the interviewees had a good experience with CPOMS as it allows easy transfer of information and communication between primary and secondary schools. However, it also takes students’ chances of starting with a clean record in secondary schools since it brings students’ previous school records to the new school. Some of these records could also be momentary problems or unnecessary opinions from their previous school teachers, which could unfairly set up a negative view of the child before the new school has a chance to form their own ideas about them.

Another significant source of information is the school information booklet, as six participants mentioned. This is a standardized booklet across all schools that provides students and parents information about the school, its ethos, and staff and resources. The booklet was expected to give students (and parents) information to familiarize themselves with the new schools. It was found essential during the COVID-19 pandemic as there were few face-to-face meetings. For instance, CM-SF-HS explained:

“The more familiar we can make the place and the more at ease we can put them at [prospective parents and pupils], the better really.”

The school information booklet is one of the two school transition projects that teachers rated as the most supportive intervention before (100% agreement) and after (90% agreement) Covid-19. Furthermore, many participants, such as HB-CA and TG-SP, were happy that the booklet was freely available and of good quality. Nevertheless, they suggested that adding a cartoon design would make it more friendly. Moreover, adding QR codes would link the booklet to online content. Finally, the data indicated that the information booklet was essential to supplement parent information evening during Covid-19.

The parent information evening helps parents explore different secondary schools and meet secondary school headteachers. According to DE-GW, the information evening allows parents to answer the question, “which school will suit my child best?”. Such information also helps the children to get adequate parental support in selecting their secondary schools. This corroborates the statement that one person’s transitions trigger the transition of significant others and vice versa (Jindal-Snape, 2016; Jindal-Snape & Cantali, 2019).

The STAR survey indicates the success of primary to secondary school transition by analyzing students’ academic and behavioral involvement in a school and their sense of belonging to the school (Rice et al., 2015; UCL, 2021). Several schools conducted the STAR survey and shared the results with teachers and parents. For example, EM-MC, DE-GW witnessed that their schools shared the STAR survey via PowerPoint presentation, and one survey result was included in the parents’ information booklet. Moreover, the participants further stated that the schools conducted additional STAR survey with parents and pupils and compared the results with the national picture. The main aim of this survey was to decrease parental concern by showing the general position of the school in relation to other schools and the national level.

“One parent had said particularly that actually she was not that worried anymore, having looked at all of the material and taking part in things, then it had really alleviated her concerns […] so that was good. And just sort of speaking to other parents, I know they found the materials useful.” (EM-MC).

Moreover, DE-GW said that the schools used the STAR survey in Personal, Social, Health and Economic (PSHE) education as a resource to talk about anxieties and initiate a discussion about the students’ expectations about secondary school.

Overall, the teachers’ views on the transition information toolkits and information evening were positive, showing a clear acceptance of the effectiveness of these projects. The teachers felt that having these projects to transfer important information across settings was valuable if executed well. Some teachers gave further suggestions on how to make these projects more effective, but agreed that they were contributing to a positive transitional experience for the children. The teachers also recognized that these projects would work well alongside current measures rather than replacing them.

#### Mental Health, Wellbeing, and Resilience Interventions

Many school transition projects aim to improve the students’ mental health, wellbeing and resilience. This paper also found that transition training, particularly ELSA, Young Minds training, SEND training, and peer mentoring, equips students to develop emotional strength to navigate the challenges they face during school transition. However, after the outbreak of COVID-19, most transition training and support were limited to online, whereas no evidence was found for the delivery of SEND training.

The data indicated that emotional literacy assistants and teaching assistants were trained in the ELSA approach to support students’ emotional needs within the schools’ resources. As AI-CNS noted, in most cases, “it is targeted provision for specific students in need, and it is one of the many tools in the toolbox.” However, some schools took a whole school approach and trained ELSA’s in every classroom to identify any difficulties students face in class and give support, which significantly improved the students’ emotional literacy. The participants also suggested that ELSA were used for students on a safeguarding plan to access extra support. JC-CAS stated:

“Based on social, emotional and mental health concerns, they will have an outcome that they need to try and achieve or attempt to achieve within those sessions. So, it might be to do with anger or stress, or you know something going on in the home and then those targets and resilience, for example, and then those targets feed into hopefully translating in the classroom.”

When talking about the children’s experiences of ELSA sessions, it was highlighted that these sessions not only build emotional resilience but also foster a sense of value and reassurance in the individual.

“Children love coming out to do the sessions, parents are really reassured that actually there is that quality support in school and staff know as well. I think they feel quite valued and reassured by it and then for all of the children, knowing that there is that kind of safety net if they need it”. (EM-MC).

Teachers showed a positive perspective on ELSA for its benefits of developing students’ emotional literacy to reduce school exclusions. ELSA is also one of the projects that continues students’ support or intervention which started in primary schools. According to AI-CNS, the hope is that the link of the intervention between primary and secondary provides a “commonality of language,” so that students with specific needs are “dealt with in a similar fashion to the way they would have been in year 6.”

“The purpose of training seminar feeder schools was that they would do ELSA in primary and then so that when they came up we could then have a link between, ELSA being done in primary and then ELSA being done in secondary” (JC-CAS).

EM-MC is one of the teachers who spearheaded the initiative in their schools. EM-MC argued that ELSA will be “one of the big legacies of the opportunity area.” Other participants such as JC-CAS and AI-CNS also asserted that ELSA had improved students’ emotional literacy and school attendance. The findings further connect ELSA to students’ social and emotional wellbeing and academic engagement (Krause et al., 2020). However, the data indicates that the COVID-19 pandemic affected some ELSA programs, prompting the support to be delivered over the phone.

The data also indicated that the Young Minds training is another intervention aimed at helping teachers understand resilience and its importance in school settings. As shown above, it is a course offered to teachers regarding mental health support for young people. However, in most cases, the training was postponed due to the COVID-19 pandemic. Moreover, the implementation of the training offered in the middle of the lockdown was unsatisfactory.

“I think the biggest issue was the fact that it was done in the middle of lockdown and so by the time we got back to school it was kind of, some of it had been semi-forgotten essentially because I think, you probably know, if you don’t apply this training straight away you usually kind of forget to don’t you, quite often?” (WG-YM)

The above excerpt indicates the impact of Covid-19, which also hindered SEND training and implementation, although 90% of the surveyed teachers thought it was essential for students’ school transition.

Peer mentoring was found to be a significant initiative in many schools. The program was designed to be bespoke to each school, but with some consistent core elements, such as legal aspects. In addition, some students were offered training on communication skills, life skill challenges, personal and social effectiveness, and other topics that help young people develop their wellbeing, confidence, and resilience. The training was initially aimed to be delivered face-to-face but was provided online due to the pandemic. Overall, peer mentoring was found to be effective with student uptake and engagement, given the circumstances.

“It was really good though because the students that participated were fully engaged. Bearing in mind it was very uncommon at that point for students to engage with professionals virtually, and yet it was quite easy for them to engage with their peers and their friends/family. I think they did well participation-wise.” (ND-EC).

However, the data also suggests that peer mentoring was not immune from Covid-19. The pandemic affected the effectiveness of the ongoing peer mentoring activities and forced the postponement of others. For example, SF-HS stated that the peer mentoring program had a “young leaders” project, where Year 12 students act as “ambassadors” and buddy up with new Year 7 students to support them, but this was suspended due to the pandemic.

Therefore, it can be argued that the teachers felt that projects associated with mental health, wellbeing, and resilience interventions had the potential to be effective in facilitating primary to secondary school transition. In addition, the teachers were keen on building emotional literacy with the children as they found it essential for wellbeing and academic achievement. However, COVID-19 had a significant impact on the implementation and effectiveness of the projects, which also affected the teachers’ perspectives on them. Therefore, the teachers suggested the need to revisit the project in post-COVID-19 times. Moreover, in line with MMT theory (Jindal-Snape, 2016; Jindal-Snape & Cantali, 2019), these transition projects, or categories, help students experience multiple transitions, such as social, academic, and emotional transitions, which also impact their colleagues.

#### School Visits and Interschool Communications

As indicated above, various transition projects simultaneously provided students with multiple transitions, as argued in Jindal-Snape’s (2016) MMT theory. For instance, some projects were explicitly aimed at the mental health and resilience of the students’ transitioning. Whereas, others worked toward providing information and strategies for collaborative working. The final group of transition projects focused on bringing primary and secondary school experiences together and fostering a strong internal communication dialogue. These projects included summer school, transitions week, interschool visits, and the Bridging Project. They draw on the importance of relationship building, with the summer school and transitions week providing students with the opportunity to access and engage in peer-to-peer and student-to-teacher relationship building. Moreover, the interschool visits and the Bridging Project created opportunities to create a connection between primary and secondary schools.

“The positive outcome [of interschool visits] has been the dialogue and the relationships, for sure, between the different sectors. I know the names of the Year 6 teachers now that teach the kids that come up. I meet them, I talk to them, I’ve got a relationship with them. That, in itself, is really important.” (HB-CA).

The data indicated that summer school, in particular, was highlighted to be “really successful” with the children who attended being “really engaged with it” (HB-CA). Another participant, AI-CNS, also stated that “the summer school got students ready for September.” AI-CNS further explained:

“The case study that for me is a young man. He spent the first two days crying nonstop. Just cried for two days and I’m not saying that glibly or as an exaggeration. It is actually what he did. We just could not stop him from crying. And by the end of the week, he said, ‘I love it here. I can’t wait to come back.’ He arrived in September and hit the ground running”.

The summer school also had the benefit of targeting activities linked with the curriculum and, therefore, familiar to the students attending the event. This was also similar to what the Bridging Project proposed to do. However, the interviewees’ mentioned some challenges surrounding the summer school, including the failure of the children to attend all the sessions and ultimately deciding which students to offer the places to.

Many schools in the sample opportunity area followed a similar format for their summer schools: (1) designed a 3-day summer school with a focus on pupil premium, SEND, or mental health affected students; (2) focused on English/Maths and mental health; (3) provided a funded summer school with a reward system; and (4) took a whole-year approach, in groups of 30 pupils, and provided some fun activities and team building. While all participating schools implemented their projects differently, the overarching feedback was of positive academic outcomes and emotional benefit to all students involved. Overall, the importance of allowing the children to explore their new schools or spaces before they are due to start is a common theme throughout all these projects. The data indicates the importance of these projects in building resilience in young people, which also corroborates other studies (Jindal-Snape, 2016; Rice et al., 2015).

The transitions week proved to be popular with schools commenting that doing this project for a whole week was beneficial to the children, compared to just 1 or 2 days as previously experienced. The transitions week help the students explore their new school environment before joining. However, the participants argued that this project works well when all schools are streamlined and attend the same dates, which was not always happening. For instance, LG-EP stated that by having a whole week the children could get any “first-day nerves” out of the way and focus more on enjoying the process thereafter.

In addition to streamlining visits across multiple schools, schools were able to lower transitional barriers through curriculum sharing actioned via projects such as the summer school and interschool visits where children could see their new spaces and complete work in their new surroundings. Moreover, the Bridging Project was effective because the children knew some of the subject content before arriving.

“Effectively, the end goal of it [the Bridging Project] was to have a piece of work that the children can take up with them to high school and they can show off to their high school teachers that are around the same topic. So when they go to their first few lessons, they already know what they’re talking about, and they’ve got that confidence.” (JS-LP)

The Bridging Project was “a project where they could be excited about things they were going to learn at secondary school in order to build their confidence.” (HB-CA). As can be seen, the cross-curriculum approach to projects where children start the work in Year 6 and continue it in Year 7 helped to bring together students from different schools and connect them through mutually understood perspectives. This approach significantly supported the children in building their personal and social effectiveness, thus facilitating a smooth school transition (Jindal-Snape, 2016; Jindal-Snape et al., 2020; Schlossberg et al., 1995).

Overall, the teachers were very complimentary about school visits and interschool communications and felt positive about their importance in building cross-site relationships. The only criticisms came from a desire to streamline the projects more to ensure the best outcome and results for the children. The teachers realized the importance of fostering strong relationships, creating an opportunity for the children to link the transition to a new setting, and building upon a curriculum they were already familiar with. The teachers also commented on the positive outcome, not only for the children but also for the teachers, in building those connections to be able to pass on to their children and support them in their transitional journey. Moreover, the data suggest that students with relevant support from their significant others, particularly teachers and parents, are more likely to experience a positive transition (Jindal-Snape & Cantali, 2019; Rice et al., 2015).

## Conclusion

This paper investigates teacher perspectives in school transitions, focusing on the role of school transition projects on resilience, behavior and academic understanding, and positive parental involvement of Year 7 students. The findings indicate that the sample opportunity area has 14 school transition projects to support students’ smooth transition, which also impacts their long-term personal and social development. These projects affect the students’ confidence, safety, wellbeing, and self-esteem, suggesting that transition is multi-dimensional (Jindal-Snape, 2016). In addition, transition not only affects the students but also significant others such as their teachers, parents and other stakeholders who also engage in the process. However, it is important to note that the schools did not apply some of the 14 projects due to various reasons such as the COVID-19 pandemic.

Overall, the 14 transition projects can be grouped into three categories or themes, based on the nature of the activities and the primary expected outputs. The first group of projects, related to transition information toolkits and information evening, are aimed to assess, identify, and share or transfer information. These projects include CTD, CPOMS, information booklets, parent information evening, and STAR Survey. Some of these projects, such as the CPOMS, also support students’ behavioral understanding and parental involvement as they allow safeguarding SEND students, logging behavior, and maintaining parental contact.

The second group of projects focuses on transition training and mentoring to improve students’ mental health, wellbeing and resilience. ELSA, Young Minds training, SEND training and peer mentoring help students develop emotional strength and experience a positive school transition process. However, these projects were severely affected by the COVID-19 outbreak (see Appendix 1). As a result, they were either postponed or conducted online in most cases. In addition, the data suggests that most of the study participants had no information about the impact of the activities undertaken online during the COVID-19 pandemic as the lockdown hindered the evaluation of the projects.

The last theme consists of transition projects focused on school visits and interschool communications to bring primary and secondary school experiences together and help students build student-to-student and student-to-teacher relationships. The summer school, transitions week, interschool visits, and the Bridging Project also provide academic support and understanding to students through targeted activities and bridging the transition from primary to secondary learning. These findings support studies arguing that communication between the primary and secondary school settings, curriculum continuity, and teacher support are essential for school transition (Coffey, 2013; West et al., 2010).

This paper implies that schools need to design various transition projects to develop students’ sense of belonging to their schools and experience positive and multiple transitions (Galton et al., 2003; Jindal-Snape, 2016). In most cases, a transition project focuses on a particular task to facilitate a successful transition from primary to secondary school by improving students’ and their significant others personal, social, emotional, and/or academic development. Nonetheless, as indicated in the above categories, there is a strong interdependence and interconnection between and among different transition projects.

Furthermore, the data revealed that several factors affect the school transition in Year 7 students. The COVID-19 pandemic, including the lockdown, was identified as the main challenge hindering the implementation of most of the transition projects and, therefore, smooth school transition. The pandemic forced schools to deliver most transition training and support their students through online platforms. Moreover, some of the school transition projects have their own limitations and side effects. The participation of students and parents is also vital to the success of school transition projects. For instance, students’ attendance was one of the challenges that affected the summer school project.

## Limitation

This paper explored teacher perspectives in school transitions during the COVID-19 pandemic. The paper is based on the perspectives of Year 7 teachers. While the data gathered cannot claim to represent the views of most school teaching staff across the sample opportunity area, we do feel they are a good representation of the key subjects arising in relation to each project and have a “robust enough” level of representation to make sound conclusions. However, this paper is not without limitations. It is based on a limited number of participants from one opportunity area. Therefore, there is a need for similar studies from different perspectives and places to strengthen the findings and broaden people’s understanding of the case. Moreover, the heavy impact of COVID-19 on the transition projects suggests the need for a further (post-COVID-19) study exploring the implementation and role of the projects.

## Appendix

**Appendix 1.** Transition Projects’ Support to Students’ School Transition Before and During the COVID-19 Pandemic.

Project	Implementation	Strongly agree (%)	Agree (%)	I don’t know (%)	Disagree (%)	Strongly disagree (%
The Bridging Project	Before After	20 10	40 30	30 50	5 10	5 0
CPOMS	Before After	45 45	35 40	10 15	10 0	0 0
Common Transfer Document	Before After	40 45	40 20	20 35	0 0	0 0
Interschool Visits	Before After	45 5	50 15	5 60	0 10	0 10
School Information Booklets	Before After	40 35	60 55	0 10	0 0	0 0
Summer Schools	Before After	40 30	55 20	5 35	0 5	0 10
STAR Survey	Before After	10 0	25 5	65 95	0 0	0 0
Peer Mentoring	Before After	30 5	50 0	15 80	5 0	0 15
Parent Information Evenings	Before After	55 5	45 50	0 30	0 5	0 10
Emotional Literacy Support Assistants	Before After	35 10	45 20	15 55	5 5	0 10
SEND Training	Before After	55 25	35 25	5 40	5 5	0 5
Young Minds Training	Before After	30 5	15 5	55 85	0 0	0 5
Transitions Week	Before After	35 5	50 20	15 55	0 5	0 15
Transition Working Group	Before After	30 15	45 35	25 45	0 0	0 5
